# Achieving a Brighter Future: A Career-Focused Mentoring Program Designed for Adolescents and Young Adults with Cancer

**DOI:** 10.5334/cie.106

**Published:** 2024-06-17

**Authors:** Sidney Henderson-Kushner, Manivel Rengasamy

**Affiliations:** 1 Connecting Champions, United States; 2 Department of Psychiatry, University of Pittsburgh, United States

**Keywords:** adolescent and young adult cancer, mentoring, needs assessment, career and school, survivorship, social determinants of health

## Abstract

Despite high survival rates for many adolescent or young adult (AYA) cancer diagnoses, the psychosocial, academic, and vocational repercussions for survivors are profound and enduring. Hospital systems are able to address many AYA needs, but the ability to provide the human connectedness and knowledge that drive long-term school and career planning is lacking. This study assessed a group of AYAs who have or had cancer (n = 108, 54% female, 71% white, mean age 20.6 ± 4.4) to determine the school, career, medical, emotional, and psychosocial needs that are not currently being met by hospital staff and support networks. We identified the most common unmet needs of AYAs, differences between needs of AYAs in active treatment and survivorship, and the role of a career-focused mentoring program developed by the nonprofit organization Connecting Champions (CC) to address the array of unmet AYA needs. We found that the most commonly reported needs were all related to career and school, and that the top needs did not differ significantly throughout the cancer journey. These findings suggest that career and school-based needs are a high priority for AYAs, yet social isolation can make the necessary people or resources inaccessible. The CC mentoring program was reported as effective in attending to unmet needs (with an average score of 95.1/100) and can be a valuable resource for hospital systems, nonprofit organizations, and health insurers to provide personalized, career-focused support to AYAs during cancer treatment and survivorship.

## Introduction

Nearly 90,000 adolescents and young adults (AYA), aged 15–39, are diagnosed with cancer in the United States each year (National Cancer Institute 2023). Additionally, there are approximately 650,000 AYA cancer survivors in the country, a number that is expected to significantly increase in the next few decades (Armenian & Chao 2024). The moment an adolescent or young adult hears the words, “You have cancer,” they are immediately at risk of a lifetime of setbacks. More than 85% of children and adolescents survive cancer, but the psychosocial, academic, physical, socioeconomic, and vocational side effects can be devastating (Brinkman et al. 2018; Janssen et al. 2022; Jones et al. 2020; Jones, Parker-Raley & Barczyk 2011; Parsons et al. 2012).

An illness as serious as cancer can force AYAs to give up on their future plans in order to focus instead on treatment, healthcare appointments, and interactions with the care team. At a time when their peers have a wealth of resources, such as internships, school counselors, and networking opportunities, young people who have or had cancer are isolated (Fardell et al. 2018). Yet, even as community resources become accessible once again upon completing treatment, AYAs are often unable to catch up to their peers, as missing a critical period in one’s adolescence or young adulthood can have paralyzing, lifelong effects (Hydeman et al. 2019; Jones et al. 2020; Parsons et al. 2012). AYA cancer survivors have significantly higher rates of unemployment, depression, and suicide, alongside lower rates of educational attainment, marriage, and quality of life (Brinkman et al. 2018; Mader, Michel & Roser 2017; Stam et al. 2006; Zheng et al. 2018). Further, the disease can plunge AYAs and their families into poverty, as a significant portion of parents of AYAs experience job loss and/or reduction of household income (Bona et al. 2014). This financial strain on families can limit support of AYAs’ career goals, including long-term goals such as college or trade school.

Despite the overwhelming long-term consequences of an AYA cancer diagnosis, little focus is given to providing individualized, career-focused connectedness and care during such an emotional and chaotic time. This attention would enhance resiliency for AYAs by supporting school and workforce readiness, increasing life satisfaction, easing psychosocial burdens, and providing a pathway to actualize career aspirations. With individualized resources and connections, AYAs can explore career paths, acquire skills and knowledge, regain self-agency, and develop actionable post-cancer plans. One method is mentoring, which has been shown to be a beneficial intervention for promoting career readiness and psychosocial well-being (Eby et al. 2008). Mentoring has the ability to boost social capital (creating social networks and community connections), which has a lifelong impact on a young person’s economic trajectory, especially those who face severe social isolation (Chetty et al. 2022). Furthermore, technology- and skills-based interventions for this population have positive effects on the ability of AYAs to cope with cancer (Zebrack & Isaacson 2012).

A valuable approach for developing an intervention that successfully responds to the needs of AYAs is user-centered design, a process that works directly with the users (in this case, the patients/participants) to develop solutions customized to them and their needs (Haines et al. 2021a). Connecting Champions (CC), a nonprofit community-based organization, utilized this approach to identify and address needs for young people with cancer that are not currently being met by hospital staff and support systems. In 2020, CC began by facilitating focus groups with AYAs. CC then integrated input from the focus groups into the development of a national career-focused mentorship program built to help young people who have or had cancer get the resources they need to achieve a brighter future. Centered around the question, “What do you want to be when you grow up?” CC pairs participants with a mentor in an associated field for a minimum of six months. Through in-person and virtual mentorship, individuals up to age 26 in treatment and survivorship have been paired with mentors from over 100 career paths (e.g. fashion, NASA engineering, various trades, and entrepreneurship). The mentors help young people explore careers, gain valuable knowledge and experience to prepare for their careers, and reclaim their identity. By leveraging technology alongside hands-on learning, the program connects young people with mentors around the world and sends supplies for participants to prepare for life beyond cancer, such as learning trade skills and starting a business from the comfort of their hospital bed or at home between rounds of treatment. There is no cost to the participant or their family in order to participate in the CC program.

In this study, we hypothesized that (1) the needs of AYAs could be identified reliably, (2) AYAs’ needs would change over time from treatment to survivorship, (3) career and school needs would be reported by a majority of AYAs, and (4) the CC mentoring program would effectively address career, school, and psychosocial needs.

## Methods

### Participants

This study included 108 AYAs in cancer treatment and survivorship; 58 females, 46 males, and 4 non-binary individuals. 71% of participants were white and 29% were individuals of color. The mean age was 20.6 years old, with a standard deviation of 4.4 years.

AYAs were referred to the CC program by 27 pediatric and adult hospitals, 8 nonprofit organizations, and 1 health insurance company. AYAs were also allowed to be referred directly from a parent or, if over the age of 18, on their own. AYAs resided in 26 states and Washington, DC.

The following information about the participants was self-reported: age at start of the CC program, gender identity, time since diagnosis, time since finishing treatment (if applicable), type of cancer, stage of cancer at diagnosis, and race/ethnicity.

### Procedure

Data collection of needs assessments and quality surveys began in March 2022 and was done as part of the design of the CC program. Participants (or their legal guardians) signed a consent form to participate in the study.

Individuals who chose to be involved in the CC program received a mentor from their career or careers of choice (e.g. fashion designer, welder). Participants then worked with their mentor for a minimum of six months. For participants interested in multiple fields, they received multiple mentors, one for each field. In addition to the mentor, a CC staff member was assigned to each participant. CC staff facilitated the mentorship by developing a personalized curriculum, as well as scheduling and attending all participant-mentor meetings. After completing an initial needs assessment, which assessed a range of needs related to daily living, career and school, medical care, and psychosocial well-being, participants chose between two mentoring programs. For participants who identified specific fields of interest, the Traditional Mentoring Program was used, facilitating a one-to-one relationship with a mentor in that field. For participants who were unsure about an intended profession, the Exploratory Mentoring Program paired them with mentors from an array of career paths to help pinpoint possible career or academic options. Follow-up needs assessments allowed CC staff to iterate the program based on how the participant’s needs changed over time.

Of note, much of CC’s programming (>90%) is virtual, with physical supplies sent to participants when hands-on learning is appropriate. For low-income and rural participants, the virtual programming creates opportunities to discover new career paths that may not be visible in their neighborhood, such as a teen from rural West Virginia whose mentor was the Director of NASA’s Jet Propulsion Lab in California. See Program Examples in the Results section and Supplementary file 1 for more detailed information on the program experience.

### Measures

#### Needs Assessments

At the start of the CC program, participants completed an adapted AYA Needs Assessment and Service Bridge (NA-SB), which assesses AYAs’ physical, psychosocial, and practical needs and then links needs to recommended hospital services (Haines et al. 2021b). Throughout the course of the mentorship, optional follow-up NA-SBs were administered every three months to assess needs over time. See Supplementary file 2 for further details of the adaptation and administration of the assessment.

#### Mentoring Processes Scale

Upon completion of the CC program, the Mentoring Processes Scale was administered to measure the AYAs’ perception of their mentoring relationship within the CC program (Tolan et al. 2020). Higher scores indicate a more positive perception. The optional measure has 52 items across five domains: role modeling and identification; advocacy; relationship and emotional support; teaching and information providing; and shared activities. Scores range from 0–100% and are based on the number of statements with which the respondent agreed. See Supplementary file 2 for further details of the administration of the scale.

### Data Analysis Process

All participants completed the initial needs assessment (n = 108), with a subset of participants completing the optional follow-up needs assessment (n = 39) and optional mentoring processes scale (n = 41). Descriptive statistics were evaluated using measures of central tendency (e.g. frequencies, means, standard deviations). T-tests were used to compare differences in the average number of needs and chi-square analyses were used to compare frequencies of individual needs items. Comparisons of the initial needs assessments were analyzed between participants who were in active treatment or finished within 12 months of starting in the CC program (n = 48) and participants who finished treatment at least five years before starting in the CC program (n = 51). For those analyses, participants who were between one and five years after completing treatment before starting (n = 9) were excluded due to a lack of meeting criteria for either group.

## Results

In this comprehensive analysis of the needs of young people in cancer treatment and survivorship, several key findings emerged, shedding light on critical aspects that have not been previously documented in research.

### Overall Needs Assessment Characterization

In the initial needs assessments, an average of 17.6 needs (n = 108, Min 0–Max 36, Sd = 10.1) were reported by individuals in the CC program, and thus participants endorsed needs on an average of 46.3% of needs items listed on the questionnaire. In a subset of participants who completed follow-up needs assessments, an average of 1.8 remaining needs (n = 39, Min 0–Max 8, Sd = 1.9) were reported, an endorsement of 4.7% of needs items.

### Most Commonly Reported Needs

**Figure 1:**
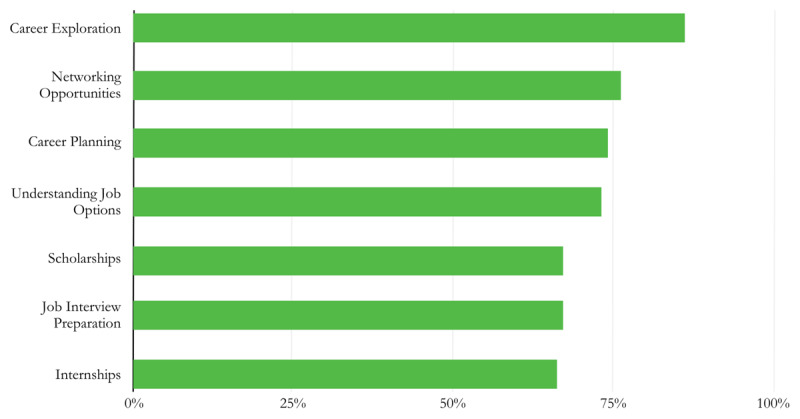
The seven most-commonly reported needs based on the initial needs assessments.

The most commonly reported needs in the initial needs assessments were all items related to school or career preparedness (see Figure 1). Seven items were reported by 66% or more participants: career experience (86% agree), networking opportunities (76%), career planning (74%), job options (73%), scholarships (67%), job interview preparation (67%), and internships (66%).

**Figure 2:**
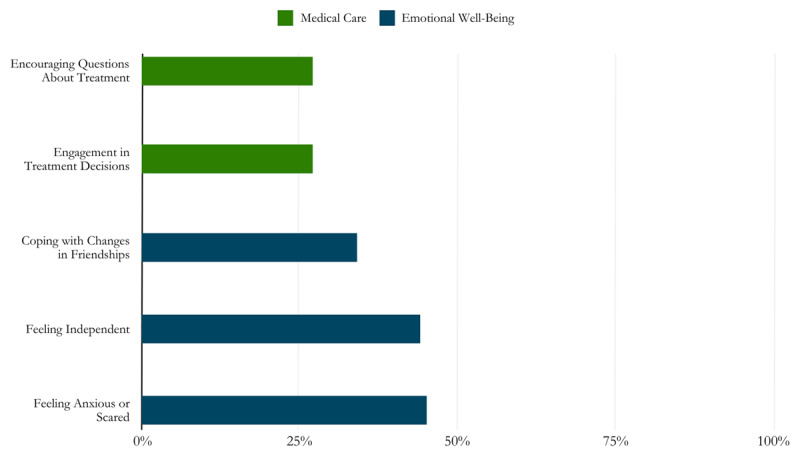
Highlighted needs related to medical care and emotional well-being based on the initial needs assessments.

Further, career and school preparedness statements were reported up to four times more frequently than statements related to cancer care or emotional well-being (see Figure 1 and Figure 2). The least reported needs in the 38-item scale were engagement in medical treatment decisions (27% agree) and encouragement to ask questions about treatment (27%). Numerous needs related to emotional well-being were reported by less than half of respondents, such as feelings of anxiety or fear (45%), lack of independence (44%), and trouble coping with changes in friendships (34%).

### Comparison of baseline needs across the treatment-survivorship spectrum

Participants (n = 108) entered the CC program at different stages in their cancer journey. Some began when they were in active cancer treatment or had finished treatment in the last 12 months (active/recent treatment group, n = 48, 54% female, 65% white, mean age 20.1 years old ± 3.8 ranging from 15–30). Other participants began when they were five or more years from finishing treatment (long-term survivorship group, n = 51, 53% female, 80% white, mean age 20.8 years old ± 4.4 ranging from 14–35). Participants who were within 1 and 5 years of completing treatment were omitted from this comparison (n = 9; see Data Analysis).

**Figure 3:**
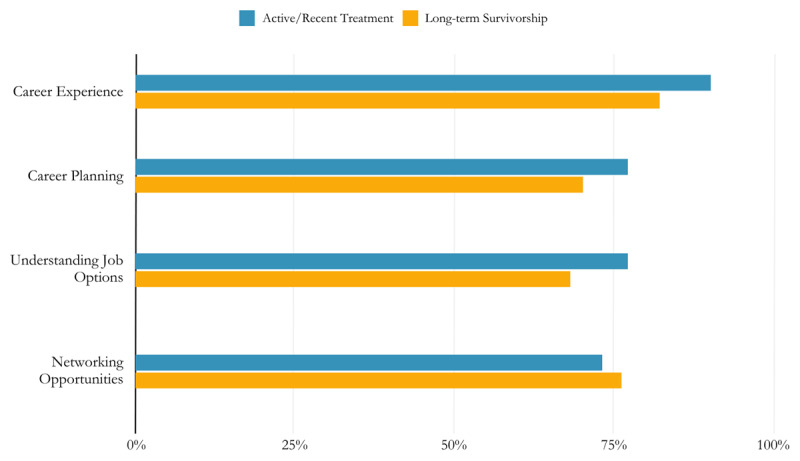
A comparison of the most commonly reported needs from treatment to survivorship from the initial needs assessments.

The active/recent treatment group reported an average of 18.9 needs and the long-term survivorship group reported 15.3 average needs, with no significant differences in needs reported between groups (t = 1.84, p > .05). For both groups, four of the top five most-reported needs were the same (see Figure 3) and proportions of participants reporting these needs did not differ by group (p’s > .05): career experience (difference between groups of 8%), career planning (7%), understanding job options (9%), and networking (3%). Feeling in control of the future was uniquely in the active/recent treatment group’s top five needs listed (i.e. not reported in the top five most-reported needs by the long-term survivorship group) and job interview preparation was uniquely in the long-term survivorship group’s top five.

For selected measures of interest, the active/recent treatment group reported the following needs more frequently: managing school life during or after treatment, X^2^ (1, N = 97) = 16.34, p < .05, managing work life during or after treatment, X^2^ (1, N = 94) = 18.77, p < .05), and feeling in control of the future, X^2^ (1, N = 98) = 5.96, p < .05.

### Perception of the mentorship experience in the CC Program

Participants perceived the mentoring relationships very positively. In a subset of participants who completed the optional Mentoring Processes Scale, an average score of 95.1 out of a possible 100 (n = 41, Min 41.7 - Max 100.0, Sd = 10.9) was reported, with higher scores reflecting more positive perceptions. For example, respondents reported that their mentor “teaches me new skills” (88% agree), “teaches me how to solve problems” (93%), and “is a good role model for me” (98%). One 20-year-old participant reported:

“My mentor is the perfect mix of helping me find solutions while also letting me learn things through experience. […] It is recognized that I learn through mistakes and I am allowed to go through them. It’s really awesome.”

Additionally, 100% of respondents reported “I want to be like my mentor when I get older.”

### Program Examples

#### Traditional Mentoring Program

A 19-year-old male in active treatment was passionate about using art to tell stories, but no longer had the same opportunities to develop new art skills and techniques after being diagnosed. In the initial needs assessment, he expressed needing additional help with 13 items, such as “Getting experience in a career field I am interested in,” “Regaining abilities I had before my treatment began,” and “Hanging out with someone who likes the same things as me.” He was paired with an art teacher who helped bring art back into his life. She met with him over video chat 1–2 times per month for approximately 11 months. Together, the participant and his mentor created an original comic book by developing characters, creating storyboards, and expressing life through art. Throughout his time in the program, he was sent supplies including a drawing light table, a sketchbook, and fine brush pens to foster hands-on learning and self-expression.

#### Exploratory Mentoring Program

A 16-year-old female in survivorship wanted to follow in her grandfather’s footsteps by practicing medicine in the US military. However, in her initial intake, she indicated she was unclear about what to do next. She wanted help figuring out what medical profession to pursue (nurse, physician assistant, or doctor), what branch of the military to join, what schools to apply to after high school, and how to accommodate restrictions she may face in the military as a recent cancer survivor. Through her 13 months in the program, she met with four mentors, one from each branch of the US military, including someone involved in the Reserve Officers’ Training Corps (ROTC) at a local college. Before each mentor meeting, she and the CC program staff member assigned to her would prepare a list of questions to ask the mentor. After each meeting, they would reflect and establish next steps as the participant gradually pinpointed her potential career path and academic goals. Following the final mentor meeting, she and her CC staff member met with a college admissions counselor to learn about and prepare for the SATs, college essays, and scholarships. Upon completion of the program, she shared the following:

“Connecting Champions helped me get a more focused plan for my future and has connected me to people I may never have been able to. I’ve changed because I now have a focused goal and I know exactly what I want to be doing in a few years.”

## Discussion

This study found that, even amidst the harshest of cancer treatments, the most commonly reported needs of AYAs with cancer—those endorsed by at least 66% of participants—were all related to career and school readiness. Importantly, such needs may be priorities for AYAs above psychosocial and emotional needs or needs related to their medical care. Career and school-related needs were endorsed by 66–86% of AYAs, whereas key needs related to emotional well-being and medical care were only endorsed by 27–45% of AYAs. Furthermore, we found that needs related to career preparedness remain unmet even long into survivorship, as no differences were found in the frequency of the top needs when comparing participants in active or recently completed cancer treatment and participants in long-term survivorship. These findings suggest that school and career needs remain a priority throughout the cancer journey, and that future-focused programs would be helpful to meet these needs for AYAs.

Participants in the CC program reported that their mentors were effective teachers and role models. With an average score of 95.1/100 for the mentoring experience, AYA reports indicate that their one-to-one connections with mentors are very valuable. By engaging AYAs in quality relationships with mentors who share their interests, the CC program provides a unique environment that empowers AYAs to change the trajectory of their future and further their long-term career, school, and psychosocial development. Our findings demonstrate that the CC career-focused mentoring program can be a beacon of support, effective at addressing high-priority needs and providing a lifeline for young people regardless of where they are in their cancer journey.

While the cost of facilitating the CC program is significant in terms of time and money, there may be opportunities for replication of the program in other countries as well. Approximately eight hours per month of staff time are required per participant (attendance at every mentor-participant meeting, preparation time for planning activities and conversations, scheduling, data collection, and administrative support) and the total cost per participant is estimated to be $3,000–$5,000 (primarily personnel costs). As the CC program is free to the participant and their family, it is essential to replication efforts that funds are dedicated from hospitals, health insurers, governments, foundations, and community members, coupled by partnerships with other non-profit organizations and workplaces.

Overall, along with prior research, our data reinforce that being removed from the community during such a vulnerable period in one’s childhood, adolescence, or young adulthood may have debilitating lifelong consequences without the appropriate interventions (Hydeman et al. 2019; Jones, Parker-Raley & Barczyk 2011). School and career support is especially critical, as it can improve AYAs’ financial security and quality of life during survivorship (Fardell et al. 2018). This type of support also complements the burgeoning legislative efforts throughout Europe around oncological oblivion (Scocca and Meunier 2022). These anti-discrimination laws establish the “right to be forgotten” for cancer survivors by removing any requirements to share medical information related to their cancer diagnosis with financial institutions or insurance companies. Leveraging legislation alongside research and interventions like the CC program fosters a more holistic approach to providing care for AYAs and survivors.

### Limitations and Future Research

While the study provides insight into a valuable intervention for this population, there are multiple limitations to consider. As with any study of hospital-based interventions, our sample size was limited considering we could not examine all hospital systems across the US. Potential sample bias may also exist given that participants were referred to the CC program. The sample size of this study is relatively small when considering the total number of young people in cancer treatment or survivorship in the general population. Further, the absence of a control group makes it challenging to attribute any reported improvements solely to the Connecting Champions mentoring program. The feasibility of a control group should be explored as the program grows, though the conceptual model of CC suggests that unmet career and school needs are prevalent.

Future directions for the program include involving a greater number of participants in the follow-up needs assessments, though preliminary results show participants are commonly reporting skill acquisition, assistance with future planning, and gained experience in a career field of interest. These early results suggest that providing mentors from the AYAs’ fields of choice for as little as six months can have a significant impact on their future. The impact of the CC program on AYAs in long-term survivorship and AYAs on government-assisted health plans is also in the process of being investigated.

Expansion of the program will be fundamental to these future analyses. Partnerships with more hospitals are critical. A broadened referral process with current hospital partners would also reach more patients. Hospital educators (alongside social workers, child/AYA life specialists, nurses, psychologists, and physicians) are some of the strongest referring partners of the organization, but an additional area of growth could be developing direct relationships with the multitude of primary, secondary, and postsecondary schools the CC program’s participants attend. The capacity for these additional relationships should be measured, as they may be especially beneficial as supplementary support for participants who indicate needing help with items related to school preparedness. Additional research should also examine the effectiveness of career-focused mentorship for adolescents and young adults who are treated in hospitals outside the US or who have life-threatening illnesses other than cancer.

## Conclusion

The world of healthcare has expertise in many fields, such as medicine, education, psychology, art, music, and social work. However, career preparedness is vitally missing. This study substantiates the vital role of career-focused mentoring programs in enhancing the lives of adolescent and young adult cancer patients and survivors. The bond AYAs develop with their mentors is especially important given their widely-reported need for and value of career knowledge, skills, and direction. Additionally, this study indicates how collaborations between hospital care teams and community-based organizations, such as Connecting Champions, can more comprehensively identify and address unmet needs of patients. The CC mentoring program has garnered widespread support from dozens of hospitals, insurance companies, and nonprofit organizations, highlighting its potential for scalability and broader implementation. For hospital staff and health insurers, the CC program has the ability to provide essential data on the unmet needs of patients/members. Collaboration and funding from hospitals, health insurers, and governments would allow for further expansion of the CC program. It would also create a sustainable, detailed roadmap on how to provide programs similar to CC at new hospitals and potentially for young people with other health conditions throughout the US and the world. By bridging the gap between medical needs and needs related to school and career, Connecting Champions has provided a model for support that extends beyond treatment. When AYAs have access to customizable resources and connections, they receive a pathway to a more promising and resilient future despite the daunting challenges of cancer.

## Additional Files

The additional files for this article can be found as follows:

10.5334/cie.106.s1 Supplementary file 1. Methods. Additional information on the program.

10.5334/cie.106.s2 Supplementary file 2. Methods. Additional information on assessments and scales.
