# Supplementary material for: Achieving a Brighter Future: A Career-Focused Mentoring Program Designed for Adolescents and Young Adults with Cancer
Source: Contin Educ. 2024 Jun 17;5(1):90–9. doi: 10.5334/cie.106 (PMC11192099; doi:10.5334/cie.106)
Supplement: Supplementary file 1. — Methods. Additional information on the program. [file cie-5-1-106-s1.pdf]

Supplementary file 1: Methods. Additional information on the program.

Detailed Procedure

Upon being referred by hospital staff or by self-referral, the adolescent or young adult (AYA) has a video intake with a Connecting Champions (CC) staff member. After the intake, they are sent the initial needs assessment. These two components allow for a qualitative and quantitative understanding of the AYA's intended career path(s) and their unmet needs. A CC staff member then finds a mentor in the community, either locally or nationally, who is in the AYA's desired field and meets the AYA's unique needs. Mentors are interviewed, trained, and required to clear necessary background checks.

Participants meet with their mentors 1-4 times per month, based on the preferences of the participant, for a minimum of six months. However, many participants meet with their mentors beyond the minimum, with some participants meeting with their mentor for upwards of three years. The average period of time in the program is approximately nine months. Duration is generally determined by whether needs remain in the optional follow-up needs assessments and the participant's desire to extend their mentorship experience. Meetings with mentors are, on average, 56 minutes in duration (n = 70, Min 28 - Max 142, Sd = 17.8). They can take place in the hospital (inpatient or outpatient), at the participant's home (for virtual meetings), at the mentor's workplace, or in the community. All meetings are supervised by a CC staff member. For virtual meetings, when relevant, physical or digital supplies are sent to both the participant and mentor to facilitate hands-on learning from the comfort of a hospital bed or home. Examples include parts to build a robot, a mannequin head and shears, video editing software, and cooking supplies.
