# Supplementary material for: Achieving a Brighter Future: A Career-Focused Mentoring Program Designed for Adolescents and Young Adults with Cancer
Source: Contin Educ. 2024 Jun 17;5(1):90–9. doi: 10.5334/cie.106 (PMC11192099; doi:10.5334/cie.106)
Supplement: Supplementary file 2. — Methods. Additional information on assessments and scales. [file cie-5-1-106-s2.pdf]

Supplementary file 2: Methods. Additional information on assessments and scales.

Needs Assessments (NA-SB)

In this study, the NA-SB was modified for the purposes of the CC program by adding 15 career and school preparedness items (e.g. understanding job options, finding networking opportunities) and 11 psychosocial items (e.g. feeling in control of my future, having a friend to rely on). Sections unrelated to the CC program, such as sexual and reproductive health, were removed. The adaptations resulted in a measure of 38 items across four domains: support systems, emotional well-being, work and education, and psychosocial needs. The original scale was also adjusted to clarify if participants need additional help in a particular area (e.g. "Agree-I want and need more help with this."). For each item, respondents indicate whether they need more help by selecting "agree", "somewhat agree," "don't know," "not right now," or "not applicable to me." The NA-SB is administered to AYAs at the beginning of each mentorship. The follow-up needs assessments were optional, in order to gather feedback from AYAs on the feasibility and appropriate frequency.

Mentoring Processes Scale (MPS)

In this study, the MPS was modified to include each original item twice: once about the mentor and once about the CC staff member (e.g. "My mentor teaches me new skills"; "My staff member teaches me new skills"). Items about the CC staff member were excluded in this study's analysis. The MPS was also optional, in order to gather feedback from AYAs on the feasibility and appropriate frequency.
